# Controllable Fabrication of Non-Close-Packed Colloidal Nanoparticle Arrays by Ion Beam Etching

**DOI:** 10.1186/s11671-018-2586-2

**Published:** 2018-06-11

**Authors:** Jie Yang, Mingling Zhang, Xu Lan, Xiaokang Weng, Qijiang Shu, Rongfei Wang, Feng Qiu, Chong Wang, Yu Yang

**Affiliations:** 1 International Joint Research Center of China for Optoelectronic and Energy Materials, School of Materials Science and Engineering, Yunnan University, Kunming, 650091 China; 2 Institute of Optoelectronic Information Materials, School of Energy Research, Yunnan University, Cuihu North Road 2, Kunming, 650091 Yunnan Province China

**Keywords:** Ion beam etching, Polystyrene nanoparticles, Nanosphere lithography, Si nanopillar arrays

## Abstract

Polystyrene (PS) nanoparticle films with non-close-packed arrays were prepared by using ion beam etching technology. The effects of etching time, beam current, and voltage on the size reduction of PS particles were well investigated. A slow etching rate, about 9.2 nm/min, is obtained for the nanospheres with the diameter of 100 nm. The rate does not maintain constant with increasing the etching time. This may result from the thermal energy accumulated gradually in a long-time bombardment of ion beam. The etching rate increases nonlinearly with the increase of beam current, while it increases firstly then reach its saturation with the increase of beam voltage. The diameter of PS nanoparticles can be controlled in the range from 34 to 88 nm. Based on the non-close-packed arrays of PS nanoparticles, the ordered silicon (Si) nanopillars with their average diameter of 54 nm are fabricated by employing metal-assisted chemical etching technique. Our results pave an effective way to fabricate the ordered nanostructures with the size less than 100 nm.

## Background

Polystyrene (PS) nanospheres have been attracting great attention from several research fields due to their ability for fabricating novel nanomaterials and nanostructures, such as ordered nanowire arrays [1–3], nanopillar arrays [4, 5], nanohole arrays [6, 7], nanodot arrays [8], core/shell composite materials [9, 10], nanomesh [11, 12], and magnetic quantum dots [13]. Particularly, nanosphere lithography has been one of the most popular research hotspots in designing the ordered nanostructure arrays since it takes the advantages of simpler process and lower cost. At the beginning of nanosphere lithography, a monolayer of PS spheres with hexagonal close-packed arrays generally forms onto a planar substrate using spin-coating method [14] or self-assembled technology [15]. After reducing the diameter of PS spheres, the non-close-packed particle arrays can produce without changing their initial position. Combined with the wet etching or dry etching technology, ordered nanostructure arrays, such as ordered Si nanopillar and Si nanohole arrays, can be fabricated [1–7, 11, 12]. The size of these nanostructures and the space among nanostructures can be well controlled by choosing different sizes of spheres and regulating the etching parameters of PS spheres.

Generally, the primary methods for reducing the diameter of PS spheres are reactive ion etching (RIE) [15–18] and plasma etching (PE) [19, 20] with a parallel plate reactor. During the RIE process, the oxygen plasma is applied to reducing the size of PS spheres. This etching rate is significantly dependent on the chemical reaction between oxygen plasma and PS. A weak physical sputtering of PS spheres is also inevitable during the oxygen plasma treatment. For the plasma etching technology, argon (Ar) plasma is employed to bombard the top surface of spheres, and the physical sputtering behavior plays an important role in this process. Both RIE and PE exhibit two characteristics of anisotropic etching because of ion bombardment [16–20]. Firstly, the shape of PS particles transforms from an isotropic sphere into a non-spherical morphology after etching. Secondly, the traverse diameter of the particles decreases nonlinearly with the increase of etching time. Furthermore, the etching rate of PS particles is very high, and the typical values of RIE and PE system are about 40–90 nm/min [6, 17, 21] and 180 nm/min [20], respectively. Thus, it is generally difficult to control the desired size of nanoparticles well below 300 nm [22]. Recently, Plettl et al. [22] and Brombacher et al. [23] develop an isotropic etching technology with a slow etching rate of 8 nm/min by using an inductively coupled plasma etching (ICPE) system. For this system, the plasma density and the bias voltage can be regulated independently, leading to the better controllability in the etching process of PS particles. Consequently, the diameter of PS nanoparticles can be well controlled to sub-50 nm. Compared with the characteristics of anisotropic etching, the nanoparticles can still maintain a spherical shape after ICPE treatment. Furthermore, a linear relationship between the traverse diameter of PS nanoparticles and the etching time is demonstrated in this isotropic etching process.

Ion beam etching (IBE) technology is also a powerful tool for fabricating various nanomaterials and nanostructures [24–26]. Different from the PE, RIE, and ICPE systems, the ion production and acceleration are separated from the substrate in the IBE system, which can avoid the bombardment of Ar plasma in the lateral direction of samples. Thus, the lateral etching of PS particles resulted from the plasma bombardment may not occur. Similar with the ICPE system, the independent regulation of ion current density and ion energy of IBE system is benefit for controlling the etching process. To the best of our knowledge, the non-close-packed arrays of polystyrene nanoparticles fabricated by using IBE have not been reported yet.

In this article, the non-close-packed arrays of PS nanospheres with the controllable diameter of sub-100 nm have been obtained after exposing to Ar^+^ ion beam with a slow etching rate. The evolution of PS nanoparticle diameters with etching time, beam current, and voltage has been studied. The effects of ion beam bombardment on the diameter reduction of PS nanoparticles have been discussed. Based on the non-close-packed nanoparticle arrays, the ordered silicon (Si) nanopillars have been fabricated.

## Methods

Polished p-type Si (100) wafers were cleaned by a standard RCA method. The PS nanospheres with the diameter of 100 nm were obtained from Alfa Company. The concentration of PS solution is 2.5 wt%. A self-assembled monolayer of PS nanospheres formed on the surface of silicon wafer by Langmuir-Blodgett approach [15]. After drying, the samples were loaded into a vacuum chamber and the background pressure was below 6.0 × 10^− 4^ Pa. The Ar gas pressure was maintained at 2.0 × 10^− 2^ Pa for current experiment. Ar^+^ ion beam was generated by a Kaufman-type ion source and bombarded the PS nanosphere film under the condition of normal incidence. The close-packed arrays of PS nanoparticles were exposed to Ar^+^ ion beam radiation at different etching parameters.

Based on a template with the non-close-packed arrays of PS nanoparticles, ordered Si nanopillar arrays were prepared by employing metal-assisted chemical etching. Firstly, a 15-nm-thick Au layer was deposited on that template by sputtering. Then, chemical wet etching was performed by immersing the samples into a mixed solution (5:1, *v*/*v*, HF/H_2_O_2_) for 1 min.

The surface morphology of PS nanoparticles was characterized by scanning electron microscope (SEM; FEI Quanta 200). The cross-sectional morphology of PS nanoparticles and the morphology of Si nanopillars were measured by field emission scanning electron microscope (FESEM; FEI Nova NanoSEM 450).

## Results and Discussion

**Fig. 1:**
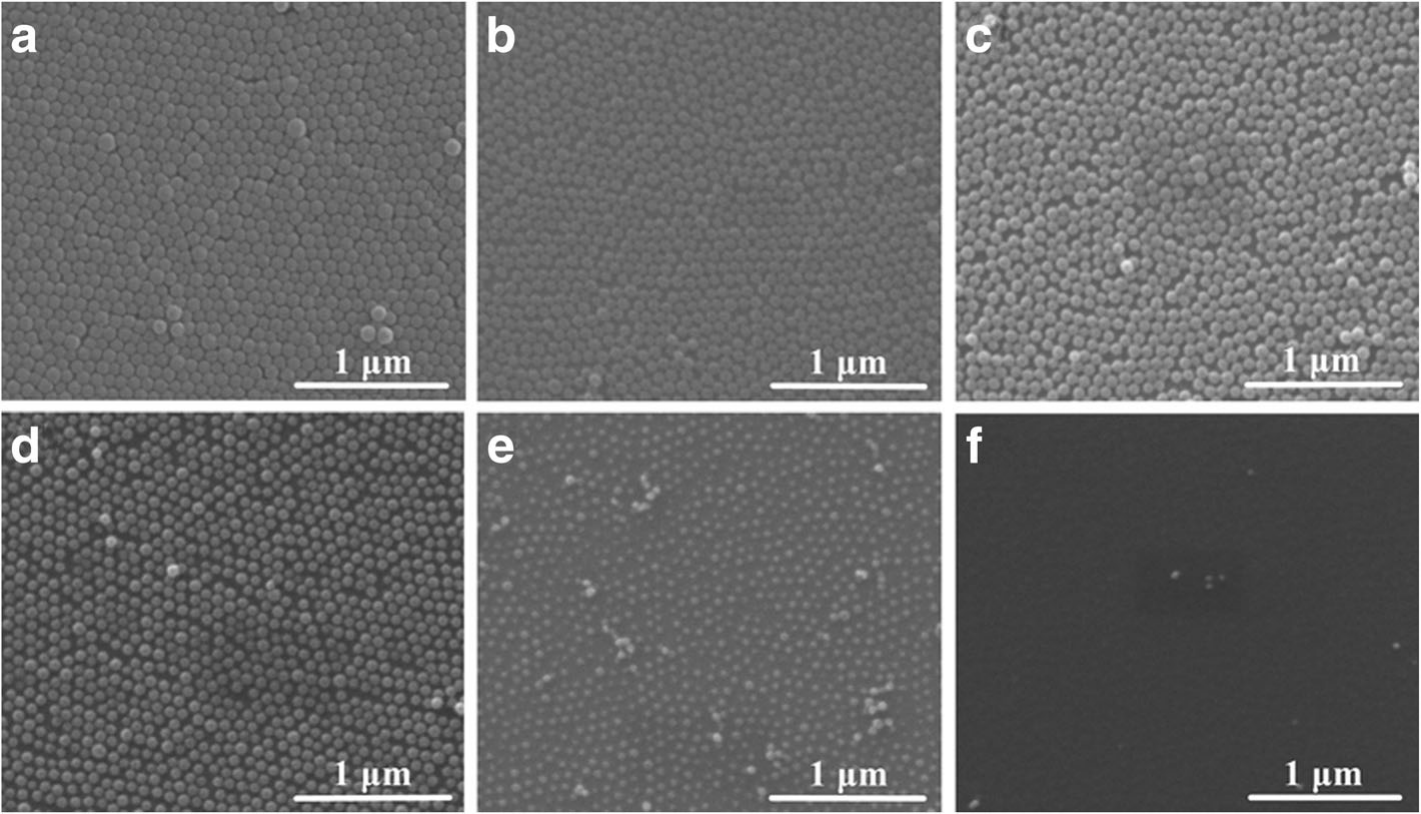
SEM images of PS nanoparticles after etching for 0 (**a**), 5 (**b**), 7 (**c**), 9 (**d**), 10 (**e**), and 11 min (**f**)

The surface morphology of self-assembled PS nanosphere film without ion beam treatment is shown in Fig. 1a. The hexagonal close-packed arrays of PS nanospheres are clearly present. Some defects, nanospheres stacked upon the arrays, are also observed simultaneously. It is generally difficult to obtain perfect monolayer of the nanospheres with the diameter of 100 nm. The PS spheres with the diameter ranging from 200 nm to several micrometers are easy to assemble into highly ordered array structures on Si wafer [1]. The reason for selecting the nanospheres with 100 nm diameter in our experiment is to compare the etching rate with that obtained by ICPE [22, 23]. It is well known that the smaller the diameter of PS particles, the higher the etching rate at the same conditions [20]. Moreover, the potential application of the ordered nanostructures with sub-100 nm diameter is attractive.

In order to discover the evolution of nanoparticle diameters with exposure time, the beam current of 3 mA and the voltage of 1 kV were selected, and the time was set as 5, 7, 9, 10, and 11 min, respectively. As shown in Fig. 1, the diameter of PS nanoparticles reduces gradually and the space among particles is enlarged with increasing the etching time. For the samples with the etching time of 5, 7, and 9 min, the range of nanoparticle diameters is about 88 ± 9, 75 ± 8, and 54 ± 8 nm, respectively. After etching for 10 min, the size uniformity of PS nanoparticles is deteriorated, and the nanoparticle diameters are about 34 ± 10 nm. After exposing to ion beam for 11 min, only a few nanoparticles distribute on the surface of Si wafer. These residual nanoparticles may come from the etching products of the defects.

**Fig. 2:**
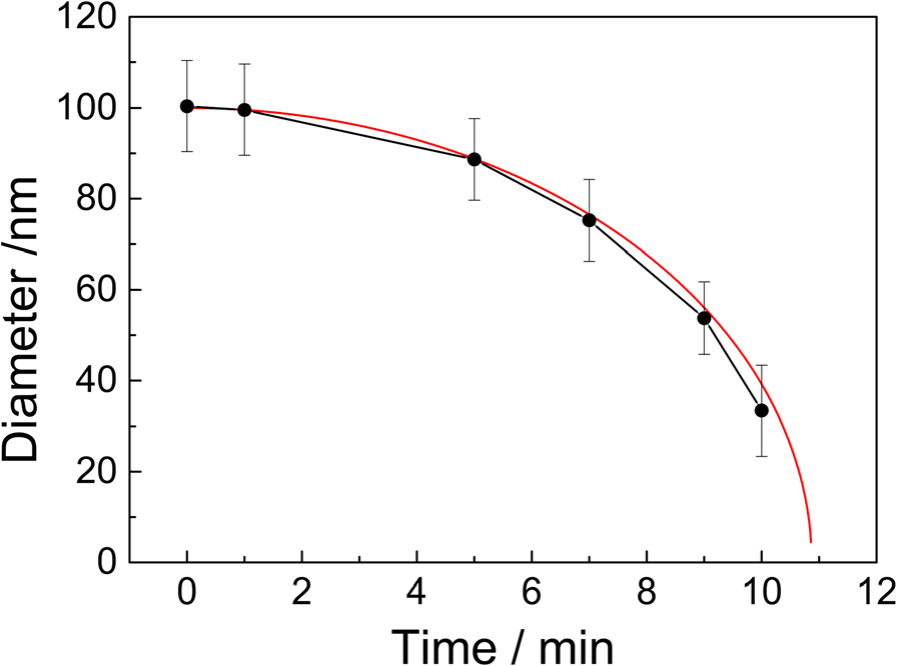
Time dependence of the reduction of traverse diameters after ion beam treatment. The dotted line is experimental data, while the red one is calculated result based on Eq. 1 with setting the *k* value as 9.2 nm/min

**Fig. 3:**
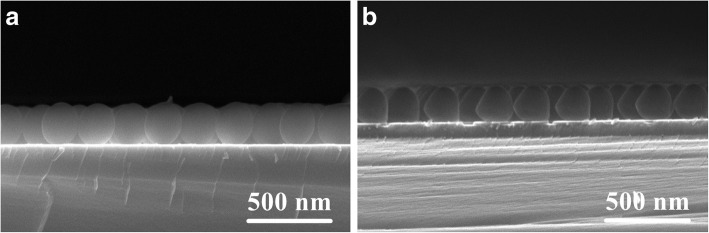
Cross-sectional FESEM images of PS nanoparticles with the diameter of 200 nm (**a**) and those after etching for 5 min (**b**). In order to clearly reflect the shape transition of nanoparticles, the nanosphere with the diameter of 200 nm was used. The shape transition of nanospheres with different initial diameters is same after exposing to ion beam

Figure 2 shows the relationship between the traverse diameter of nanoparticles and the etching time. A nonlinear reduction of the traverse diameter with increasing the etching time is observed. This trend is one major characteristic of anisotropic etching technology and similar with that of previous works prepared by RIE and PE [16–20]. Furthermore, another characteristic of anisotropic etching technology can also be seen in Fig. 3. Comparing the cross-sectional morphology of the particles without etching with that after exposing to ion beam for 5 min, a shape transition of PS particles from a sphere to a non-spherical morphology is obviously observed. Since Ar^+^ ion beam bombards the top surface of PS particles under the condition of normal incidence, where the physical sputtering will occur preferentially. The lateral etching of PS particles resulted from the bombardment of Ar plasma may not occur due to a separation of ion production and acceleration from the samples. The etching rate in the longitudinal direction of the particles is higher than that in the traverse direction. A difference between etching rates in two different directions induces the anisotropic etching of PS nanoparticles. As a result, the longitudinal diameter of non-spherical particles is smaller than their traverse diameter. The cross-sectional shape of non-spherical particles looks like an ellipse, while the surface morphology of non-spherical particles is still circle. In addition, Tan demonstrated that the etching of PS particles along the longitudinal direction was uniform with increasing the etching time for RIE technology [17]. Thus, the etching rate is usually defined as the reduction of the longitudinal diameters per unit time [17, 20]. Based on the shape transition of particles, the etching rate along the longitudinal direction can be calculated as follows [20]:

1 $$ D=\sqrt{4{R}_0^2-{k}^2{t}^2} $$

where *D* is the traverse diameter of PS particles, *R*_0_ is the radius of initial PS nanosphere, *k* is the etching rate along the longitudinal direction, and *t* is the etching time. According to Eq. 1, the etching rate at the exposure time of 5, 9, and 10 min is calculated to be about 9.2, 9.3, and 9.4 nm/min, respectively, in our experiment. These values are smaller than those obtained from RIE [17, 21] and PE [20], while they are close to those values achieved from ICPE [22, 23]. It is suggested that IBE technology has a greater potential to better control the etching process of PS nanoparticles because of their slow etching rate.

Moreover, it is also noticed that the etching rate is non-uniform with increasing the time. In Fig. 2, as the traverse diameter of nanoparticles is further reduced to less than half of its initial value, the experimental points fall below the theoretical value calculated based on Eq. 1 with setting the *k* value as 9.2 nm/min. This indicates that the etching rate increases at longer etching time. The evolution is different from the results which mainly depended on the chemical reaction between oxygen plasma and polystyrene (such as RIE and ICPE) [16–18, 22, 23]. A similar tendency was also reported by Cao et al. [20] using the PE technology recently. They proposed that the higher etching rate at longer exposure time was attributed to the occurrence of lateral etching. However, the lateral etching of PS particles resulted from the plasma bombardment may not occur in IBE system. We suppose that the thermal energy accumulated gradually during the physical bombardment of accelerated ions may have a significant impact on the etching rate at longer exposure time. An increase of the etching rate of PS particles has been proved by Plettl et al. [22] after annealing at 75 °C. It is well known that a part of kinetic energy of the accelerated ions will be converted into the thermal energy of samples after ion beam treatment. Okuyama and Fujimoto [27] demonstrated that the target could be heated up to 2000 °C after Ar^+^ ion bombardment if the target had poor heat conduction. Actually, most of the thermal energy can be taken away from the water-cooled target. But the temperature of samples placed on the target with water or gas cooling is still kept in a range of 70–150 °C after a long-time treatment of ion beam [28, 29]. When the substrate temperature is higher than 135 °C, PS nanospheres could melt and be adhesive together [30]. This phenomenon is not observed in our samples, which indicates that the temperature will not exceed 135 °C during the process of ion beam bombardment. Therefore, the increase of the etching rate at longer exposure time may be ascribed to the thermal effect of ion bombardment. At this time, the etching of PS nanoparticles is determined by the physical sputtering and thermal effect together.

To our knowledge, the hexagonal non-close-packed arrays of PS particles adhered to Si wafer cannot be purchased from markets. One possible reason is that the arrays fabricated by using RIE and/or ICPE are easily separated from Si wafer. In order to compare the fastness of non-closed-packed arrays fabricated by ICPE with that of the arrays prepared by IBE, two samples with similar nanoparticle diameters and periodicity were produced by ICPE and IBE system, respectively. After immersing in 2.5% HF solution for 3 min and then rinsing with de-ionized water, the nanoparticles in the sample prepared by ICPE disappear, while the nanoparticles in the sample fabricated by IBE still adhere to the surface of Si wafer without changing their periodicity. It is indicated that the fastness of PS nanoparticles prepared by IBE is better due to the thermal effect of ion beam bombardment. For further application, the nanoparticles can be removed by immersing into dichloromethane solution for 2 h. These results suggest that the non-close-packed arrays of PS particles prepared by employing IBE have a great potential to promote the commercial application of nanosphere lithography. And the non-close-packed arrays may be available from markets in future.

**Fig. 4:**
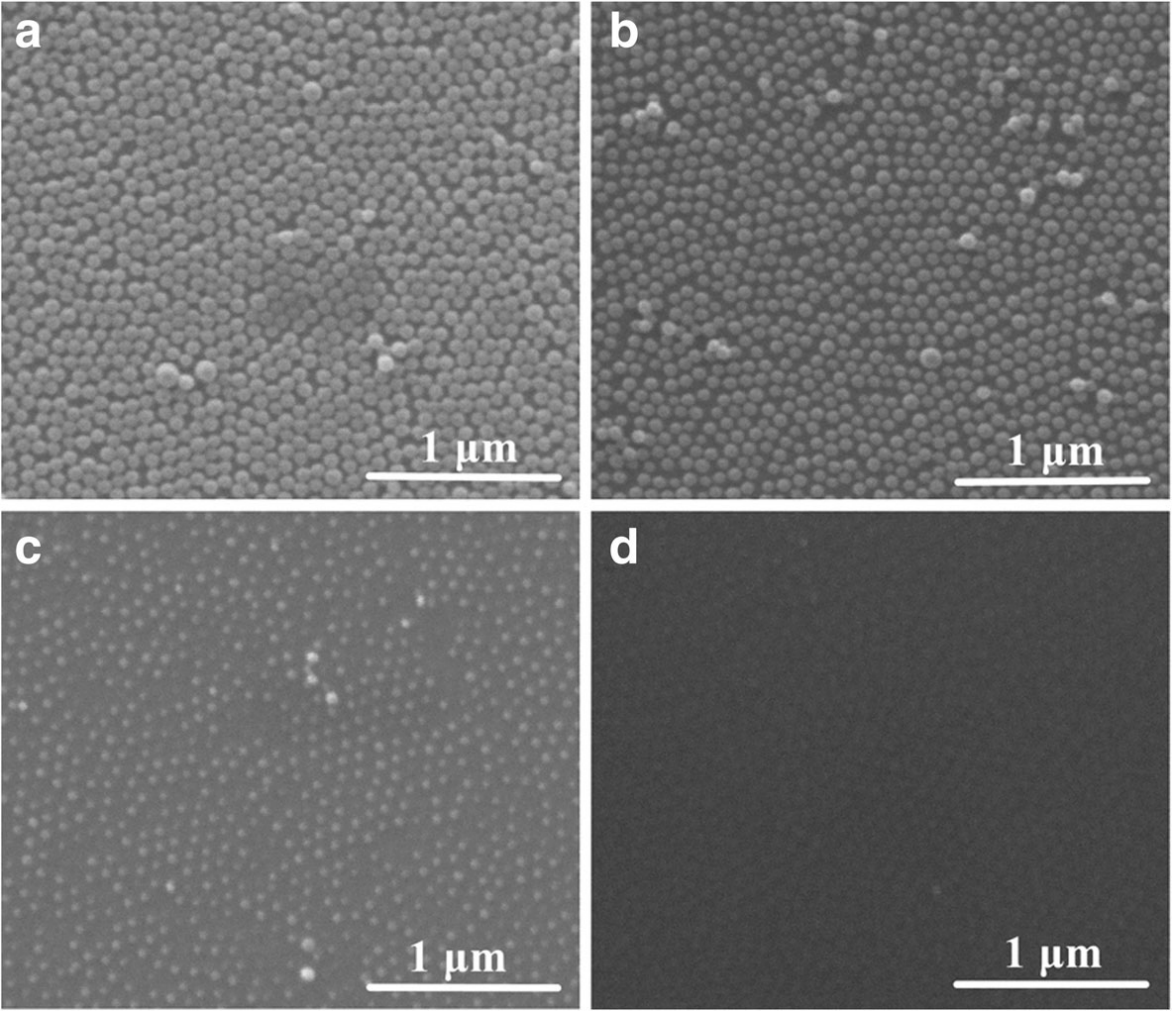
SEM images of PS nanoparticles etched for 5 min with the ion voltage of 1 kV and the beam current of 5 (**a**), 7 (**b**), 9 (**c**), and 10 mA (**d**), respectively

Beam current is also an important factor to regulate the etching rate in IBE. The diameter reduction of PS nanoparticles exposed at different beam currents (3, 5, 7, 9, and 10 mA) is discussed. As shown in Fig. 4, the diameter of nanoparticles decreases with increasing the beam current. At the current of 10 mA, no PS particle is observed, but the surface of Si wafer is not smooth. Many small islands, of which the periodicity is similar with that of the PS nanoparticle arrays, distribute on the surface (Fig. 4d). It is suggested that both Si substrate and PS particles can be etched by Ar^+^ ion beam without selectivity. In contrast with the surface roughness of sample prepared at a current of 3 mA for 11 min (Fig. 1f), the roughness is larger at the current of 10 mA for 5 min in Fig. 4d. This suggests that the damage of Si substrate is serious at larger beam current.

**Fig. 5:**
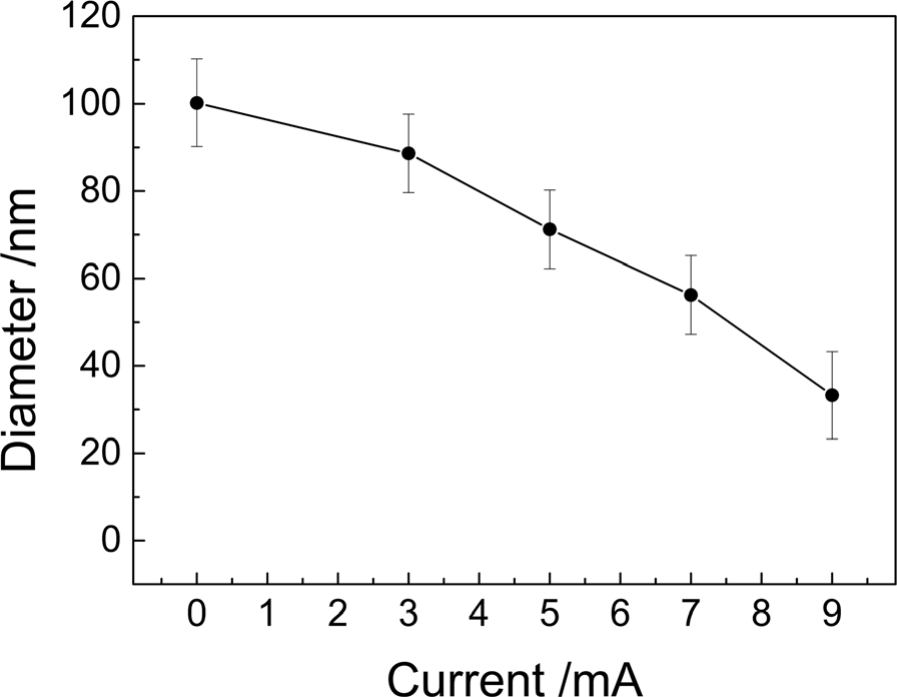
Beam current dependence on the etched nanoparticle diameter

The beam current dependence on the nanoparticle diameter is shown in Fig. 5. A nonlinear reduction of nanoparticle diameters is present with the increase of beam current. This is similar with the evolution of diameter with increasing the power in RIE and PE system [16–22]. The etching rate is about 18.9 nm/min at the current of 9 mA.

**Fig. 6:**
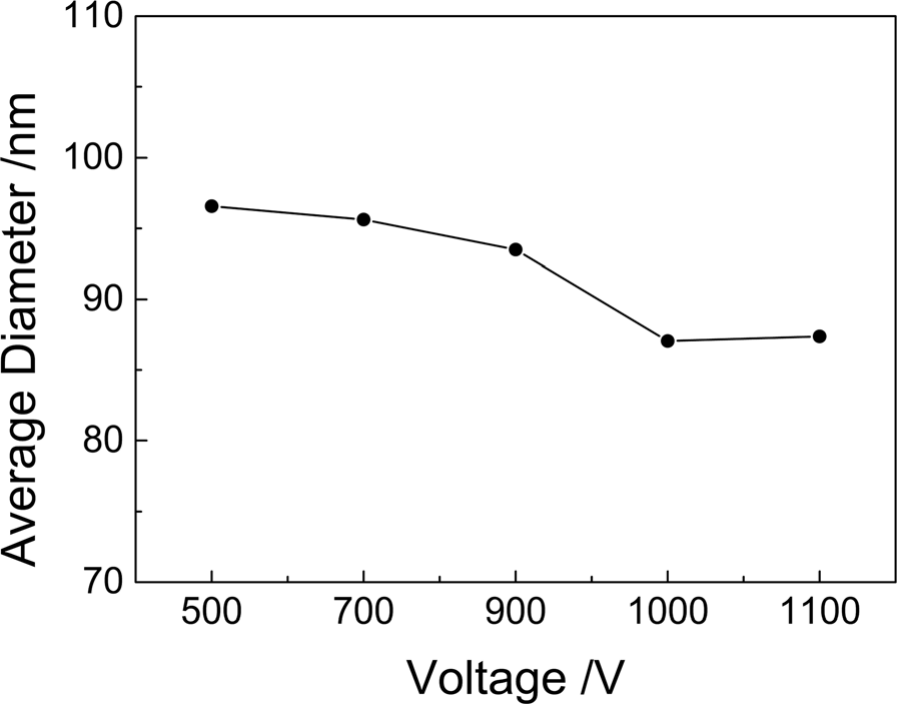
The average diameter of nanoparticles as a function of beam voltage with the ion current of 3 mA and the etching time of 5 min

The kinetic energy of accelerated Ar^+^ ions is determined by beam voltage. The effect of beam voltage on the diameter reduction is also investigated. The beam voltage was set as 500, 700, 900, 1000, and 1100 V respectively. With the increase of beam voltage, the diameter of nanoparticles reduces slightly. In Fig. 6, a little decrease of average diameter is observed with increasing the beam voltage. When the voltage is larger than 1 kV, the etching rate remains stable.

**Fig. 7:**
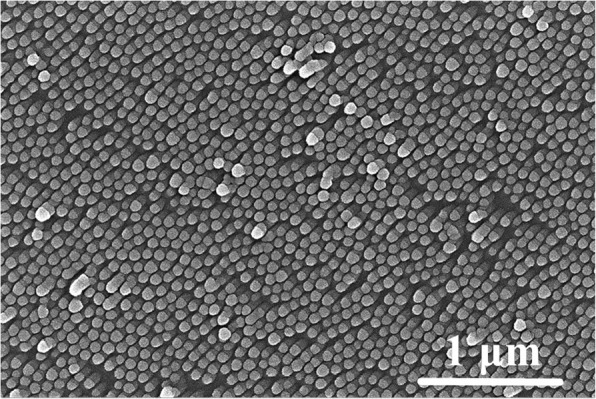
FESEM image of Si nanopillar arrays

Based on the template with non-close-packed arrays of PS nanoparticles prepared by using IBE, ordered Si nanopillar arrays were fabricated by employing metal assisted chemical etching. The morphology of Si nanopillar arrays is shown in Fig. 7. The average diameter and height of Si nanopillar are about 54 nm and nearly 100 nm, respectively. On the top of Si nanopillars, PS particles still exist.

## Conclusions

The monolayer of PS nanospheres with the diameter of 100 nm was exposed to Ar^+^ ion beam. The hexagonal non-close-packed arrays of PS nanoparticles with the controllable diameter in the range from 34 to 88 nm were fabricated on Si substrate. The evolution of particle diameters with exposure time, beam current, and voltage were well studied. With increasing the exposure time, the traverse diameter of nanoparticles decreases nonlinearly. At the period of long etching time, the etching rate increases obviously, this results from the thermal effect of ion beam bombardment. With the increase of beam current, the etching rate increases from 9.2 to 18.9 nm/min. The slow and controllable etching rate is beneficial to control the desired size of nanoparticles below 100 nm. Based on the template of non-close-packed arrays of PS nanoparticles, the ordered Si nonopillars were fabricated by using metal-assisted chemical etching. Furthermore, the better fastness of the nanoparticles prepared by using IBE exhibits a great potential application in nanosphere lithography.

## Abbreviations

FESEM: Field emission scanning electron microscope
IBE: Ion beam etching
ICPE: Inductively coupled plasma etching
PE: Plasma etching
PS: Polystyrene
RIE: Reactive ion etching
SEM: Scanning electron microscope
